# Mapping the murine TMJ glenoid fossa over development, homeostasis and in response to disease

**DOI:** 10.1111/joa.70068

**Published:** 2025-11-10

**Authors:** Ticha Tuwatnawanit, Denisa Belisova, Zuzana Sumbalova Koledova, Neal Anthwal, Abigail S. Tucker

**Affiliations:** ^1^ Centre for Craniofacial and Regenerative Biology, Faculty of Dentistry, Oral and Craniofacial Sciences, King's College London London UK; ^2^ Department of Conservative Dentistry and Prosthodontics Faculty of Dentistry, Srinakharinwirot University Bangkok Thailand; ^3^ Department of Histology and Embryology Faculty of Medicine, Masaryk University Brno Czech Republic; ^4^ Laboratory of Tissue Morphogenesis and Cancer, Institute of Molecular Genetics of the Czech Academy of Sciences Prague 4 Czech Republic

**Keywords:** bone remodelling, fibrocartilage, FSP1, osteoarthritis, temporomandibular joint

## Abstract

The temporomandibular joint (TMJ) plays a key role in facilitating complex mammalian jaw movements required for daily life. It is formed between the condylar process of the mandible (or dentary bone) of the lower jaw, the glenoid (or mandibular) fossa of the squamosal/temporal bone in the upper jaw and an interposed fibrocartilage disc. Structural defects in any component of the TMJ can disrupt the entire joint, contributing to TMJ disorders. Embryonic defects in the condyle in mice have been shown to have an impact on the shape and development of the glenoid fossa, highlighting the importance of coordinated development of the two sides of the joint. Although recent research has focused on the condylar process, much less is known about the development and homeostasis of the glenoid fossa, and defects in the glenoid fossa are also evident in disease. Here, we have analysed the formation and molecular identity of the glenoid fossa using the mouse as a model. Our findings revealed distinct patterns of development of the fossa in the anterior, middle and posterior regions. Interestingly, the cartilage marker Sox9 was transiently expressed in the lateral branch of the glenoid fossa during early TMJ development, and the loss of *Sox9* in *Wnt1‐cre;Sox9fl/fl* mice resulted in the absence of this part of the fossa. Postnatal maturation of the murine glenoid fossa was marked by the initiation of a fibrocartilage layer, the formation of which coincided with the onset of independent feeding, suggesting a role for mechanical force in glenoid fossa fibrocartilage induction. In contrast to the condyle, the fossa fibrocartilage expressed low levels of FSP1, a marker of the stem/progenitor population of the condyle. Depletion of FSP1‐positive cells by conditional diphtheria toxin activity in *FSP1‐Cre;DTA* mice has previously been shown to cause a severe TMJ osteoarthritis phenotype and enlargement of the condylar head postnatally. Interestingly, here, we show that in reaction to changes in condylar shape, these mutants develop an increase in glenoid fossa angulation over time, associated with increased remodelling activity, particularly in the lateral branch of the fossa. These findings highlight that the fibrocartilage of the glenoid fossa and condyle are not equivalent and that changes in the condyle can have a knock‐on secondary effect on the 3D structure of the fossa. This coordinated response would allow for alignment of the TMJ, maintaining function throughout life, even in the case of disease.

## Graphical abstract

The fibrocartilage of the murine glenoid fossa and condyle of the temporomandibular joint are shown to form at distinct times with distinct molecular identities. Changes in the condyle during disease lead to remodelling of the fossa to maintain joint alignment.

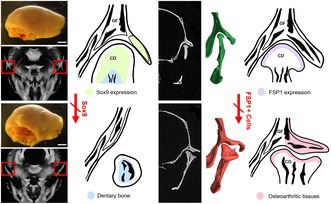

## 1 INTRODUCTION

The temporomandibular joint (TMJ), often known as the jaw joint, is a ginglymoarthrodial joint that facilitates the intricate jaw movements essential for daily life in mammals (Bender et al., 2018; Wadhwa & Kapila, 2008). An evolutionary novelty in mammals (Anthwal & Tucker, 2022), the TMJ forms the connection between the upper and lower jaws forming an articulation between the condylar head of the dentary bone and glenoid fossa (or mandibular fossa) of the squamosal/temporal bone (Murphy et al., 2013). Within this skeletal framework, a fibrous articular disc, known as the TMJ disc or meniscus, serves as a cushion between the articulating surfaces (Wadhwa & Kapila, 2008). Any structural, functional and physiologic dysregulations of the muscles or osseous components involving the TMJ are called temporomandibular disorders (TMDs), which affect 34%–38% of the general population (Minervini et al., 2023; Zieliński et al., 2024). Degeneration of the TMJ can lead to TMJ osteoarthritis (TMJOA), characterised by bone structure modifications and articular cartilage degradation (Cardoneanu et al., 2022). Patients with TMJOA face chronic pain, crepitus, a severely limited range of motion and reduced quality of life (Acri et al., 2018).

While most research on degenerative TMJ changes has focused on the condylar process, defects in the glenoid fossa are also observed in TMJOA (Bechtold et al., 2016; Justina et al., 2022). Evaluating degenerative modifications in the glenoid fossa region is equally important for effective clinical management. However, the development and homeostasis of the TMJ glenoid fossa have been less extensively studied when compared to the formation of the condylar cartilage (Wang et al., 2011; Yasuda et al., 2012). The glenoid fossa, part of the squamosal bone, develops through intramembranous ossification (Liang et al., 2015), in contrast to the condyle, which forms as a secondary cartilage that undergoes endochondral ossification (Anthwal et al., 2008). Previous research in rats indicates that the glenoid fossa was identifiable at embryonic day (E) 15.5, with the anterior portion remaining unossified at E19.5, and ossification completing by E20.5 (Yamaki et al., 2005). The development of the glenoid fossa in the mouse TMJ has been shown to begin at E14.5 (Liang et al., 2015).

The TMJ articular surfaces are protected by fibrocartilage rather than hyaline cartilage, which cushions the knee and hip joints (Temenoff & Mikos, 2000). Fibrocartilage is a specialised load‐bearing tissue composed of both fibrous and cartilaginous components, allowing it to withstand tensile and compressive forces (Singh & Detamore, 2009; Stocum & Roberts, 2018). The fibrocartilage surfaces of the condylar head and the glenoid fossa in the adult transfer residual stress to the underlying trabecular bone and produce synovial fluid to lubricate the joint (Stocum & Roberts, 2018; Utreja et al., 2016). Condylar fibrocartilage has a distinct molecular signature, with high levels of fibroblast‐specific protein 1 (FSP1) expressed during the postnatal stage (Tuwatnawanit et al., 2025). FSP1‐positive cells contributed to the formation of the entire condylar skeleton, and their destruction led to severe TMJOA (Tuwatnawanit et al., 2025).

The development of the glenoid fossa has been suggested to depend on the presence of the underlying condyle, with embryonic defects and disease pathology in the condyle of mice impacting the shape and development of the glenoid fossa secondarily (Bechtold et al., 2016; Wang et al., 2011). Disruption to the fossa has also been shown to impact the formation of the disc due to displacement of muscles (Li et al., 2021), while changes to the lateral pterygoid and temporalis muscles in *Spry1* and *Spry2* mutant embryos were shown to lead to loss of the glenoid fossa (Purcell et al., 2012; Yang et al., 2015). Taken together, these studies emphasise that tissue interactions in the TMJ are critical for the development of an effective joint.

This study aims to clarify the developmental progression of the glenoid fossa by analysing its formation and genetic identity using a mouse model. Through 3D reconstructions and immunofluorescence staining, we demonstrated that the glenoid fossa matures postnatally, forming a fibrocartilaginous layer that is molecularly distinct from the fibrocartilage of the condyle. In the *FSP1‐cre;DTA* mouse model, the loss of fibrocartilage in the condyle resulted in significant defects in TMJ morphology and structure, accompanied by remodelling of the glenoid fossa. These findings highlight the coordinated response that enables the alignment of the TMJ under disease conditions.

## 2 MATERIALS AND METHODS

### 2.1 Animal preparation

*FSP1‐Cre* mice (Bhowmick, 2004) were mated to *R26mTmG* or *R26RDTA* (referred to as *DTA*) (Ivanova et al., 2005) mice to allow lineage tracing of the FSP1‐expressing population of cells and to generate transgenic mice for lineage depletion studies. All mating was carried out under the approval of the Ministry of Education, Youth and Sports of the Czech Republic (license # MSMT‐24093/2021‐3), supervised by the Expert Committee for Laboratory Animal Welfare of the Faculty of Medicine, Masaryk University, at the Laboratory Animal Breeding and Experimental Facility of the Faculty of Medicine, Masaryk University (facility license 310 #58013/2017‐MZE‐17214). Transgenic animals were maintained on a C57BL/6 background. *FSP1‐Cre;DTA+/− mice* were used as the mutant group and *FSP1‐Cre‐negative;DTA+/−* littermates were used as controls. In *FSP1‐Cre;mTmG* mice, *FSP1*‐expressing cells and their lineage expressed GFP (green fluorescent protein), while non‐*FSP1*‐expressing cells expressed RFP (red fluorescent protein).

*Wnt1‐Cre;tdTom*, *Wnt1‐Cre;Sox9fl/+*, *Sox9fl/fl* and CD1 mice were bred at King's College London, under UK Home Office licenses and regulations in line with the regulations set out under the United Kingdom Animals (Scientific Procedures) Act 1986. *Wnt1‐Cre;Sox9fl/+* male mice were mated with *Sox9fl/fl* female mice (Wang et al., 2011), and pregnant females were collected at E18.5 of the pups. Additional wildtype (CD1) mice were collected at a range of prenatal and postnatal stages (E14.5–E18.5, P1, P10, P14, P17, P28). *Wnt1‐Cre* MGI:2386570 and *Sox9fl* MGI:2385468 were used. For details of other lines, see Tuwatnawanit et al. (2025). All mice were housed in individually ventilated or open cages, all with an ambient temperature of 22°C, a 12 h:12 h light:dark cycle and food and water ad libitum.

### 2.2 Microcomputed tomography scanning, 3D reconstruction and analysis

Tissue samples were fixed in 4% paraformaldehyde at 4°C overnight and then washed with phosphate‐buffered saline (PBS). Skull samples were placed into a 9 or 19 mm tube (dependent on age/size) and scanned using a SCANCO MEDICAL μCT 50 scanner (70 kV, 114 μA, 20 μm). The TMJ was reconstructed in 3D using Amira and MeshLab software.

Glenoid fossa angulation was analysed following set landmarks (Figure S1): (1) The most caudal point of the suture between the squamosal and greater wing of the sphenoid bone; (2) The join between the squamosal body and the zygomatic process of the squamosal; (3) The most caudal point of the suture between the zygoma (jugal) and zygomatic process of the squamosal bone (Richtsmeier et al., 2000).

### 2.3 Tissue preparation

**FIGURE 1:**
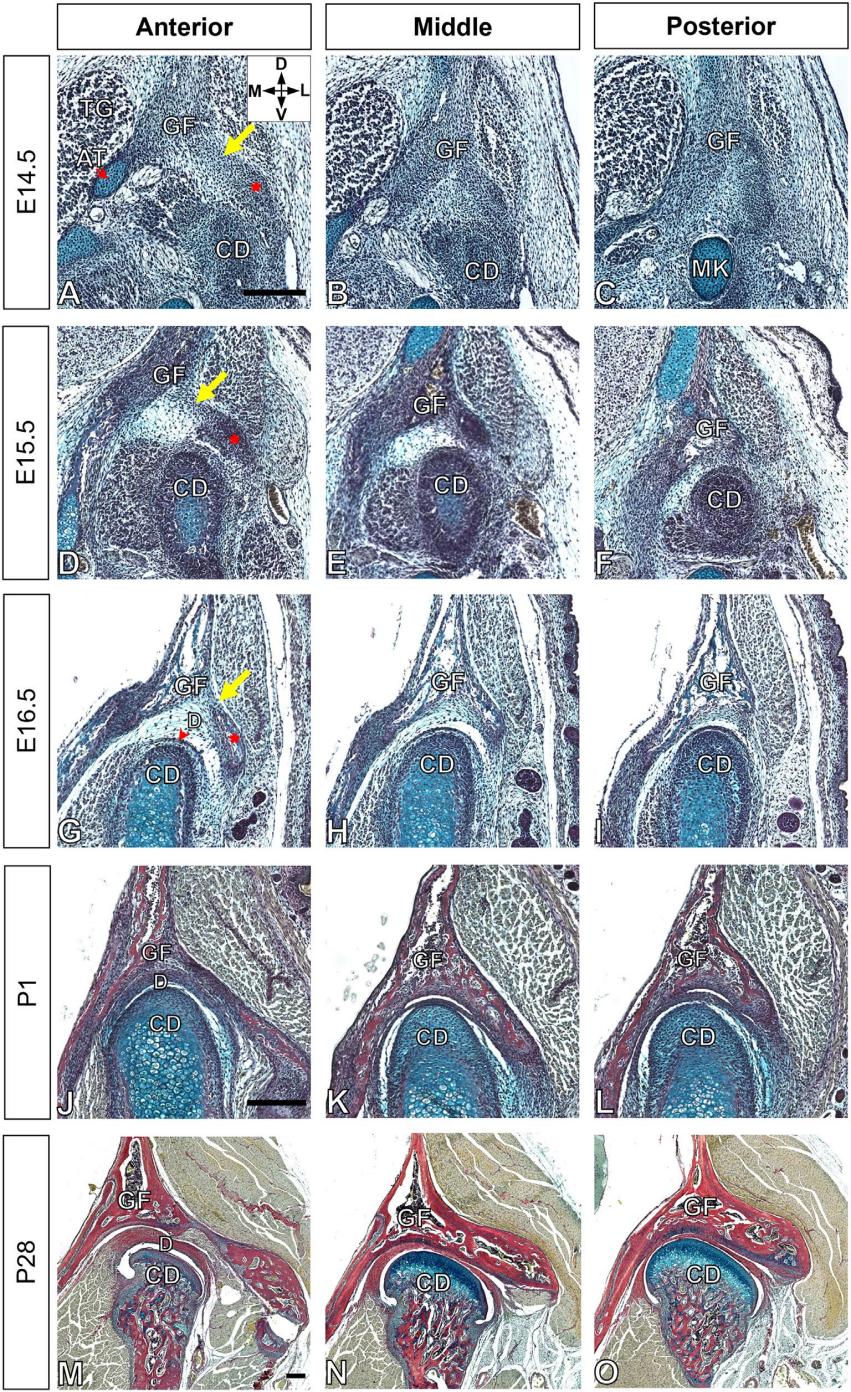
Development and growth of the mouse TMJ. (a–o) Picrosirius red–alcian blue trichrome staining illustrating the development of the TMJ during embryonic stages at E14.5 (a–c), E15.5 (d–f) and E16.5 (g–i), and during postnatal growth at P1 (j–l) and P28 (m–o) in CD1 mice. Each stage includes images showing the anterior, middle and posterior aspects of the TMJ in frontal sections. The yellow arrow points to a fibrous tissue band between the zygomatic process and the squamosal bone. The red asterisk marks the lateral branch of the glenoid fossa. (g) The red arrowhead points to the TMJ disc. Data are representative of five independent biological replicates. The scale bar represents 200 μm. The scale bar in (a) applies to (b)–(i), in (j) to (k) and (l), and in (m) to (n) and (o). AT, Ala temporalis; CD, condyle; D, dorsal; D, TMJ disc; E, embryonic stage; GF, glenoid fossa; L, lateral; M, medial; MK, Meckle's cartilage; P, postnatal stage; TG, Trigeminal ganglion; V, ventral.

Postnatal tissue samples were decalcified in 0.5 M ethylenediaminetetraacetic acid (EDTA) pH 8 at room temperature (RT), dehydrated through a graded ethanol series, embedded in paraffin and sectioned at 6 μm in the frontal plane using a Leica RM2245 microtome (Leica Biosystems). All figures use tissue sections oriented as shown in Figure 1a. Sections were then mounted sequentially on Superfrost Plus slides (Epredia) for embryonic tissue or TruBOND380 slides (Matsunami) for postnatal tissue.

### 2.4 Trichrome staining

Serial tissue sections of the TMJ were stained using standard trichrome staining protocols for histological investigation by GEMINI AS (Thermo Scientific; ref. A81500001). The staining procedure utilised 0.5% Sirius red, 10 mg/mL Alcian blue and Ehrlich haematoxylin to distinguish various tissue components (Liang et al., 2015). Glycosaminoglycans in cartilage were stained blue (Alcian blue), collagen in fibrocartilage and bone were stained red (Sirius red), and nuclear chromatin was stained by Ehrlich haematoxylin, without staining the cytoplasmic structures. After staining, the slides were mounted using Neo‐Mount® (Sigma‐Aldrich; ref. 1.09016.0100) and covered with glass coverslips. A NanoZoomer Digital Slide scanner 2.0‐HT (Hamamatsu Photonics) with NDP.view2 software was used to examine and capture the morphology of histology‐stained TMJ slides.

### 2.5 Tartrate‐resistant acid phosphatase (TRAP) staining

Paraffin‐embedded slides were first deparaffinised using Neo‐Clear (Sigma‐Aldrich, Lot HX14932543, ref. 1.09843.5000). The slides were then rehydrated through a series of decreasing ethanol dilutions. Following rehydration, the sections were incubated with a TRAP‐specific substrate solution containing Naphthol AS‐TR phosphate (Sigma, Lot#SLCC8157), dissolved in N,N‐dimethylformamide (Sigma‐Aldrich, Lot#STBD6849V) and sodium L‐(+)‐tartrate dihydrate (Alfa Aesar, Lot 10168938) and dissolved in acetate buffer at pH 5.2. Within osteoclasts possessing TRAP activity, the TRAP enzyme hydrolysed the substrate, resulting in the release of phosphate. The solution also contained Fast Red TR Salt (Sigma‐Aldrich, Lot#MKBF9253), a diazonium salt that facilitates the staining of TRAP‐positive cells. Sections were incubated at 37°C for 30 min in the TRAP substrate solution. The released phosphate combined with the diazonium salt, forming a red‐coloured precipitate. After incubation, the sections were washed with double deionised water. Haematoxylin was applied as a counterstain. Finally, the stained sections were mounted onto glass slides using a mounting medium (Aquatex; Lot HC860590, ref. 1.08562.0050), coverslipped and observed under a light microscope (Nikon Eclipse 80i). Osteoclasts or other TRAP‐positive cells appeared as red‐stained cells or areas against a contrasting background.

### 2.6 Immunofluorescence and RNAscope

Immunofluorescence staining was performed for FSP1, Sox9, type II collagen (Col‐II), PCNA, type X collagen (Col‐X), GFP and RFP (Table S1). Following deparaffinization with Neoclear and rehydration through a graded ethanol series, antigen retrieval was carried out by incubating paraffin sections in trypsin at RT for 10 min. Tissue sections were then enzymatically treated with chondroitinase ABC (0.1 unit/mL) and hyaluronidase (0.6 unit/mL) for 45 min at 37°C. To minimise non‐specific binding, sections were blocked for 30 min at RT using a standard blocking buffer (1% bovine serum albumin, 0.1% Triton X‐100 in PBS). Primary antibodies, diluted in blocking buffer, were applied overnight at 4°C in a moisture chamber. The following day, slides were washed and incubated with secondary antibodies for 2 h at RT in the dark. Nuclei were counterstained with DAPI (Fluoroshield™ Sigma‐Aldrich).

RNAscope® in situ hybridization was conducted using the RNAscope Multiplex Fluorescent Detection Reagents Kit v2 (Advanced Cell Diagnostics) according to the manufacturer's protocol. *Axin2* probes were used (Table S1), and negative control staining was performed (Figure S2). Imaging was carried out using a confocal microscope (ZEISS LSM 980), and experimental data were analysed with ImageJ.

### 2.7 Statistical analysis

All experiments were conducted in at least three independent replicates. Statistical analysis was carried out using GraphPad Prism 10. Comparisons among the groups were assessed using one‐way analysis of variance (ANOVA) or two‐way ANOVA followed by Tukey's post hoc test. To compare the control and mutant groups, an unpaired *t*‐test was used. Data are presented as mean ± standard deviation (SD), with statistical significance set at *p‐*values < 0.05.

## 3 RESULTS

### 3.1 Development of the TMJ glenoid fossa from embryology to postnatal stages

The fossa, as a 3D structure, varies in morphology dependent on position along the anterior–posterior axis, with the fossa joining to the zygomatic process anteriorly. The fossa was, therefore, analysed at its anterior, middle and posterior as it formed from E14.5 to postnatal day 28 (P28). Five mice were analysed at each stage, with limited morphological variation observed between individuals.

At E14.5, the TMJ first appeared as a series of mesenchymal condensations. The glenoid fossa was located adjacent to the trigeminal ganglion and ala temporalis (which forms the greater wing of the sphenoid bone). It was positioned superior to the condyle and Meckel's cartilage (Figure 1a–c). Notably, the glenoid fossa exhibited a primary branch with a less distinct region connecting the lateral branch, forming a triangular shape that became progressively narrower from anterior to posterior (Figure 1a–c). The mesenchyme between the glenoid fossa and condyle became denser before the two primordial components of the TMJ were separated by the TMJ disc. By E15.5, the mesenchymal condensation of the condyle had increased in size and acquired an ovoid shape. At this stage, ossification of the glenoid fossa started from the posterior part and progressed towards the anterior part (Figure 1d–f). As they progressed towards each other, the condyle fitted into the concave shape of the glenoid fossa. The gap between the developing condyle and glenoid fossa remained broad. Ossification of the glenoid fossa was already visible in the anterior region at E16.5 (Figure 1g), between the zygomatic process and the squamosal bone, with a fibrous tissue band in between. In the posterior section, the zygomatic process merged with the squamosal bone to create the glenoid fossa (Figure 1i). Additionally, the superior joint cavity became clearly discernible at E16.5 (Figure 1g–i). The rapid expansion of the condyle and glenoid fossa resulted in a reduction of the joint space. The major structures of the TMJ were identifiable at E17.5, and by E18.5, the formation of the inferior joint cavity had occurred (Figure S3).

During the early postnatal period P1, a cluster of hypertrophic chondrocytes persisted at the centre of the condylar head. The process of endochondral ossification in the condyle had progressed (Figure 1j–l), while the glenoid fossa had ossified (Figure S4). TMJ development continued postnatally as the mouse jaw grew to maturity (Figure S5). By P28, the condylar head exhibited a distinct mushroom‐like morphology, accompanied by the development of a fully mature bone structure in the glenoid fossa, with a layer of alcian blue cells at the articulation surface of the fossa (Figure 1m–o).

### 3.2 Transient Sox9 expression during early TMJ development was required for glenoid fossa initiation

**FIGURE 2:**
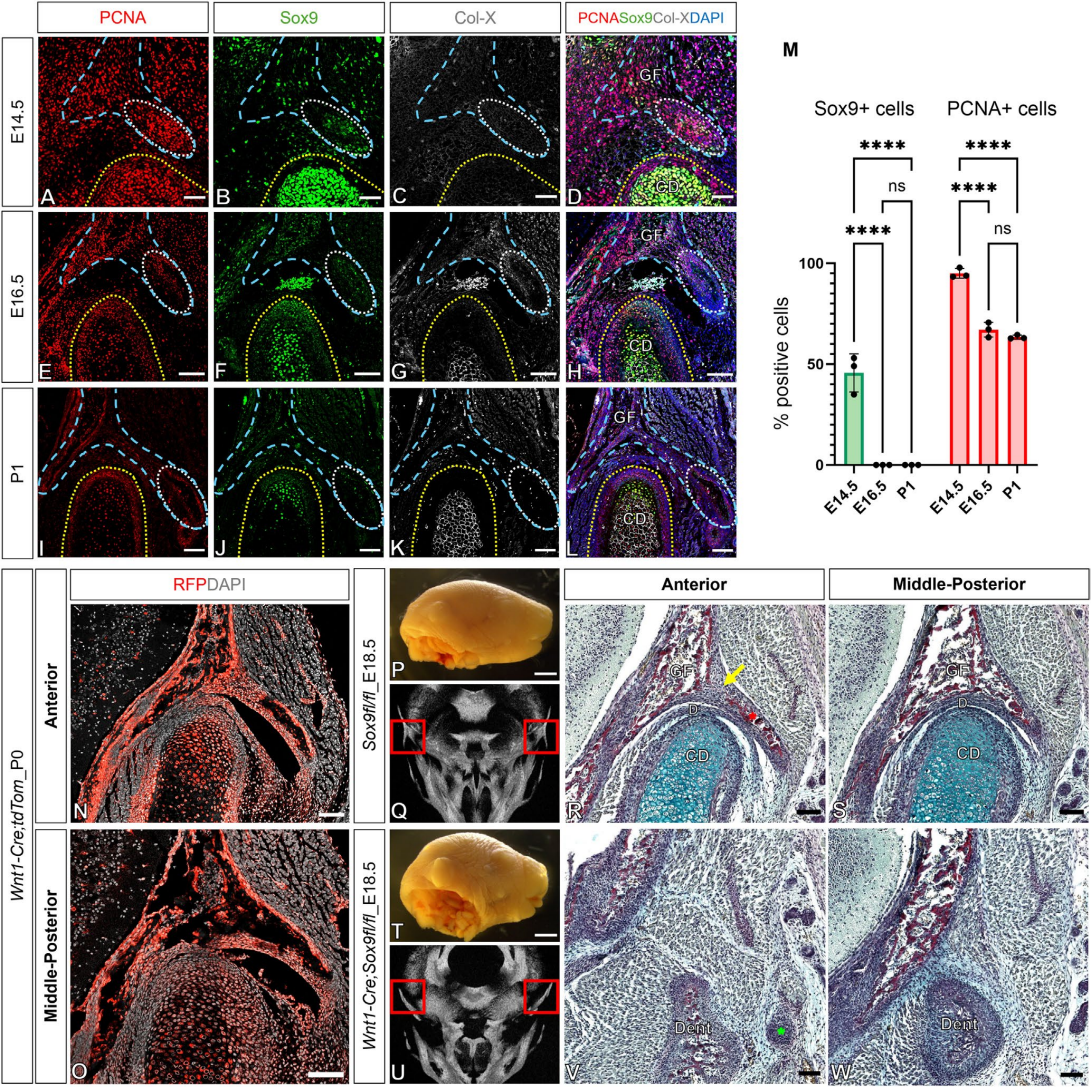
Transient Sox9 expression at the lateral branch of the glenoid fossa and absence of this part of the fossa in neural crest‐specific *Sox9* conditional knockout mice. (a–l) Immunofluorescence staining for PCNA (red), Sox9 (green), Col‐X (grey) and DAPI (blue) in E14.5, E16.5, and P1 CD1 mice. Each stage includes images showing the anterior aspects of the TMJ in frontal sections. The yellow dot line indicates the condylar head. The cyan line indicates the glenoid fossa region with the white circle marking its lateral branch. (m) Quantification of Sox9‐positive cells and PCNA‐positive cells during glenoid fossa growth. Error bar = ±SD; *n* = 3; two‐way ANOVA followed by Tukey's multiple comparisons test. (****p < 0.0001). (n, o) Immunofluorescence staining for RFP (red) and DAPI (grey) in *Wnt1Cre;tdTom* at P0 in anterior and middle‐to‐posterior aspects of the TMJ. (p) *Sox9fl/fl* mice (control) at E18.5. (q) μCT scan of the skull of *Sox9fl/fl* mice with the red box highlighting the TMJ region. (r, s) Picrosirius red–alcian blue trichrome staining illustrating the TMJ at E18.5 in *Sox9fl/fl* mice, including images showing the anterior and middle‐to‐posterior aspects of the TMJ in frontal sections. The yellow arrow points to a fibrous tissue band between the zygomatic process and the squamosal bone. The red asterisk marks the lateral branch of the glenoid fossa. (t) *Wnt1‐Cre;Sox9fl/fl* mice (mutant) at E18.5. (u) μCT scan of the skull of *Wnt1‐Cre;Sox9fl/fl* mice with the red box highlighting the absence of the lateral branch of the glenoid fossa (absent in 3/3 mutants). (v, w) Picrosirius red–Alcian blue trichrome staining illustrating the absence of TMJ formation in mutant mice compared to the equivalent region in Cre‐negative DTA littermate controls at E18.5 (absent in 3/3 mutants). All data are representative of three independent biological replicates. The green asterisk marks the zygomatic arch. (r, v) Anterior; (s, w) middle‐to‐posterior aspect of the TMJ. Scale bars: (a–d) 50 μm; (e–l, n,o, r, s, v, w) 100 μm; (p, t) 5 mm. Dent, dentary bone.

Sox9, a key transcription factor in chondrogenesis and endochondral ossification, has been shown to be expressed before the onset of osteogenic markers in the developing skeleton, including areas that undergo intramembranous ossification (Akiyama et al., 2005; Anthwal et al., 2015). At E14.5, the forming TMJ exhibited strong proliferation, marked by high expression of PCNA (Figure 2a,d). Sox9 was expressed at high levels in the condyle at this stage, but interestingly expression was also observed in the condensing mesenchyme of the forming lateral branch of the glenoid fossa (Figure 2b,d). Sox9 can be expressed by endothelial cells during development (Fuglerud et al., 2022). We, therefore, checked whether the Sox9‐positive cells co‐expressed the endothelial marker CD31. Interestingly, some colocalization of Sox9 and CD31 was evident in the fossa at E14.5; however, Sox9 in the lateral branch was not associated with the vasculature (Figure S6). Sox9 in the fossa gradually diminished over time, correlating with a decrease in PCNA expression (Figure 2e–m). Notably, no Col‐X expression, a marker of hypertrophic chondrocytes, was detected in the fossa, in contrast to the condyle which was undergoing endochondral ossification (Figure 2c,g,k). The region of the fossa is at a border between neural crest and mesoderm‐derived parts of the skull (McBratney‐Owen et al., 2008). To assess whether the entire mesenchyme of the fossa was formed from neural crest lineage tracing was performed using *Wnt1‐Cre;tdTom* mice. This confirmed that the mesenchyme of the fossa, including the lateral branch, was neural crest‐derived (Figure 2n,o). The role of *Sox9* in the fossa was then investigated by knocking out *Sox9* in neural crest‐derived cells using *Wnt1‐Cre;Sox9fl/fl* mice. The conditional mutants exhibited a shorter lower jaw and a failure of both the condyle and lateral branch of the glenoid fossa to form in all mutant mice compared to Cre‐negative *Sox9fl/fl* controls (Figure 2p–w) (3/3 mutants). μCT imaging and trichrome staining of these embryos at E18.5 clearly highlighted the absence of the lateral branch (Figure 2u–w). Interestingly, the *Wnt1‐Cre;Sox9fl/+* (heterozygous) mice also showed a mild phenotype with thinning of the lateral branch of the fossa (Figure S7). Sox9 is, thus, essential for the correct formation of the fossa.

### 3.3 Initiation of the fibrocartilage of the fossa occurs postnatally coinciding with the onset of independent feeding

The condyle is formed from a secondary cartilage, which provides the fibrocartilage layer at the site of articulation. In contrast, the fossa forms from membranous bone, which needs to be lined with fibrocartilage in order to create an articulation surface. These two processes are thus very different. The development of the fibrocartilage layer of the glenoid fossa was followed by assessing the expression patterns of chondrogenic markers (Sox9 and COL‐II, which indicate chondrocyte differentiation) and the fibrochondrocyte marker (FSP1) (Tuwatnawanit et al., 2025).

**FIGURE 3:**
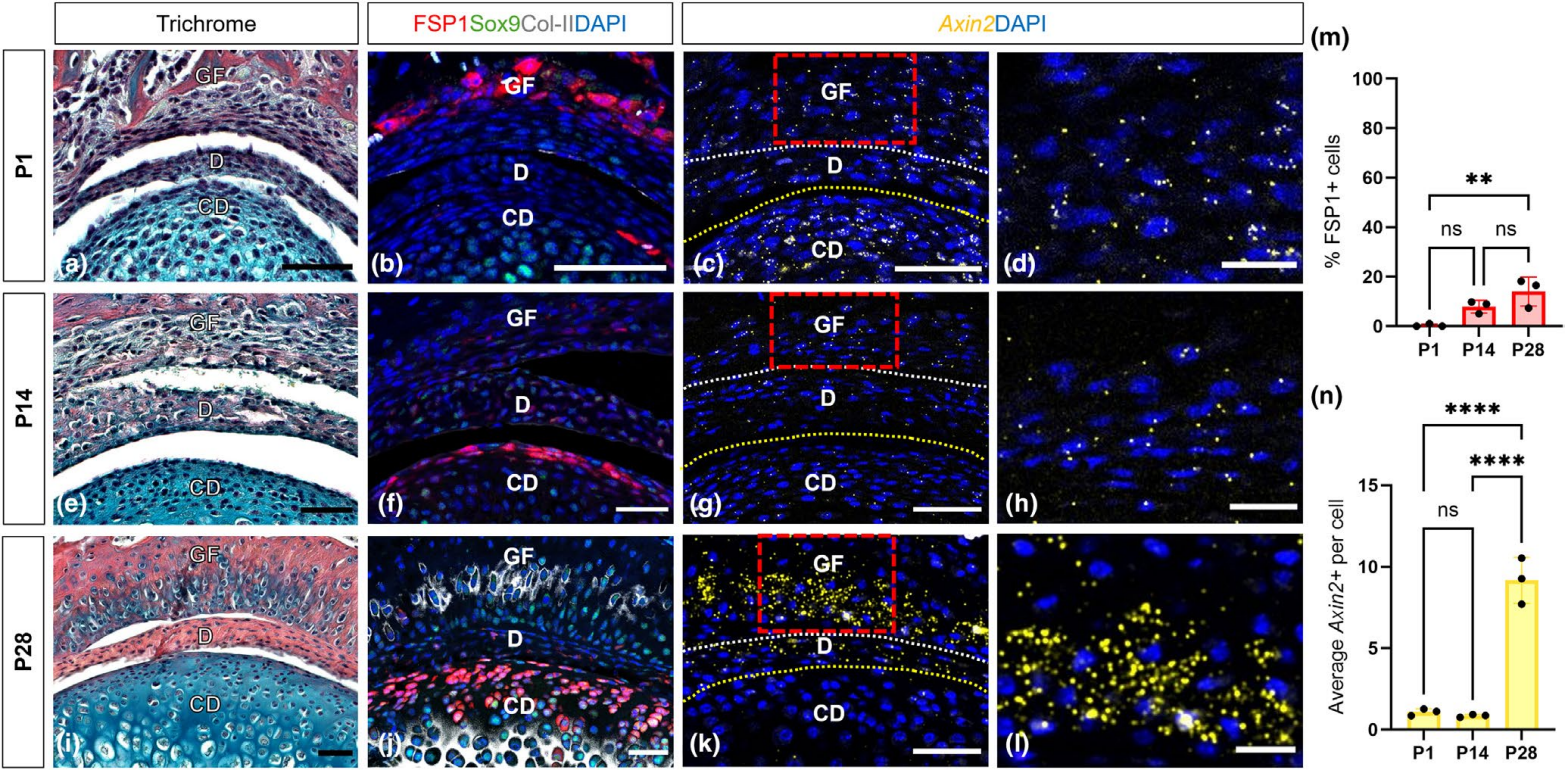
Fibrocartilage development in the postnatal glenoid fossa. (a, e, i) Picrosirius red–alcian blue trichrome staining illustrating the development of fibrocartilage in the TMJ. Scale bar: 50 μm. (b, f, j) Immunofluorescence staining for FSP1 (red), Sox9 (green), Col‐II (grey) and DAPI (blue) illustrating differences in the fibrocartilage layer between the condyle and the glenoid fossa. Scale bar: 50 μm. (c, g, k) RNAscope staining for *Axin2* expression (yellow) and DAPI (blue) in the TMJ. The yellow dot line indicates the condylar head. The white dot line indicates the glenoid fossa. Scale bar: 50 μm. (d, h, l) Magnified images showing *Axin2* expression (yellow) and DAPI (blue) in the fibrocartilage layer of the glenoid fossa (indicated by red boxes in c, g and k). Scale bar: 20 μm. (m, n) Quantification of FSP1‐positive cells and average *Axin2‐*positive cells during glenoid fossa growth. Error bars represent ±SD; *n* = 3; statistical analysis was conducted using one‐way ANOVA followed by Tukey's multiple comparisons test (**p < 0.01, ****p < 0.0001).

Embryonically and around birth (P1), there was no histological or molecular evidence of a fibrocartilage layer in the glenoid fossa (Figure 3a–d, Figure S8A). Sox9‐positive cells were exclusively located in the chondroblastic zone of the condyle, with no Sox9 or Col‐II detected in the presumptive fibrocartilage layer of the glenoid fossa during the early postnatal stage. Only a few FSP1‐positive cells were scattered around the condyle and the ossified region of the glenoid fossa at this stage (Figure 3b, Figure S8A). Following tooth eruption around P14, FSP1 expression was localised to the superficial surface of the condyle but, in contrast, only a few cells showed weak expression in the glenoid fossa (Figure 3e,f).

After weaning at P28, Sox9 and Col‐II were detected in the prechondroblastic and chondroblastic zones of the condyle, respectively (Figure 3i,j). At this stage, robust expression of Sox9 and Col‐II was observed in the superficial layer of the glenoid fossa (Figure 3j), aligning with the alcian blue expression observed in trichrome staining of this region (Figure 3i). These findings suggest that the development of the fibrocartilage layer in the glenoid fossa occurs during the juvenile stage, coinciding with the functional onset of the TMJ at weaning. Interestingly, despite the robust expression of FSP1 in the condyle, the glenoid fossa exhibited very few FSP1‐positive cells on the articular surface (Figure 3j,m). To further assess the relative contribution of FSP1‐expressing cells to the condyle and fossa, lineage tracing was performed using *FSP1‐Cre;mTmG* mice. Agreeing with recently published data, the adult condyle was populated by FSP1‐lineage cells, while the articulating surface of the fossa was almost completely absent of FSP1‐lineage cells (Figure S8B,C). FSP1 expression in the condyle has been shown to be negatively regulated by *Axin2*, a readout of canonical Wnt activity (Tuwatnawanit et al., 2025). In keeping with this finding, in contrast to the condyle, high levels of *Axin2* persisted in the FSP1‐negative fossa (Figure 3c,d,g,h,k–n). The fibrocartilage of the fossa and condyle, therefore, have different modes and timing of development and distinct molecular signatures.

### 3.4 Loss of FSP1‐positive cells led to glenoid fossa remodelling

**FIGURE 4:**
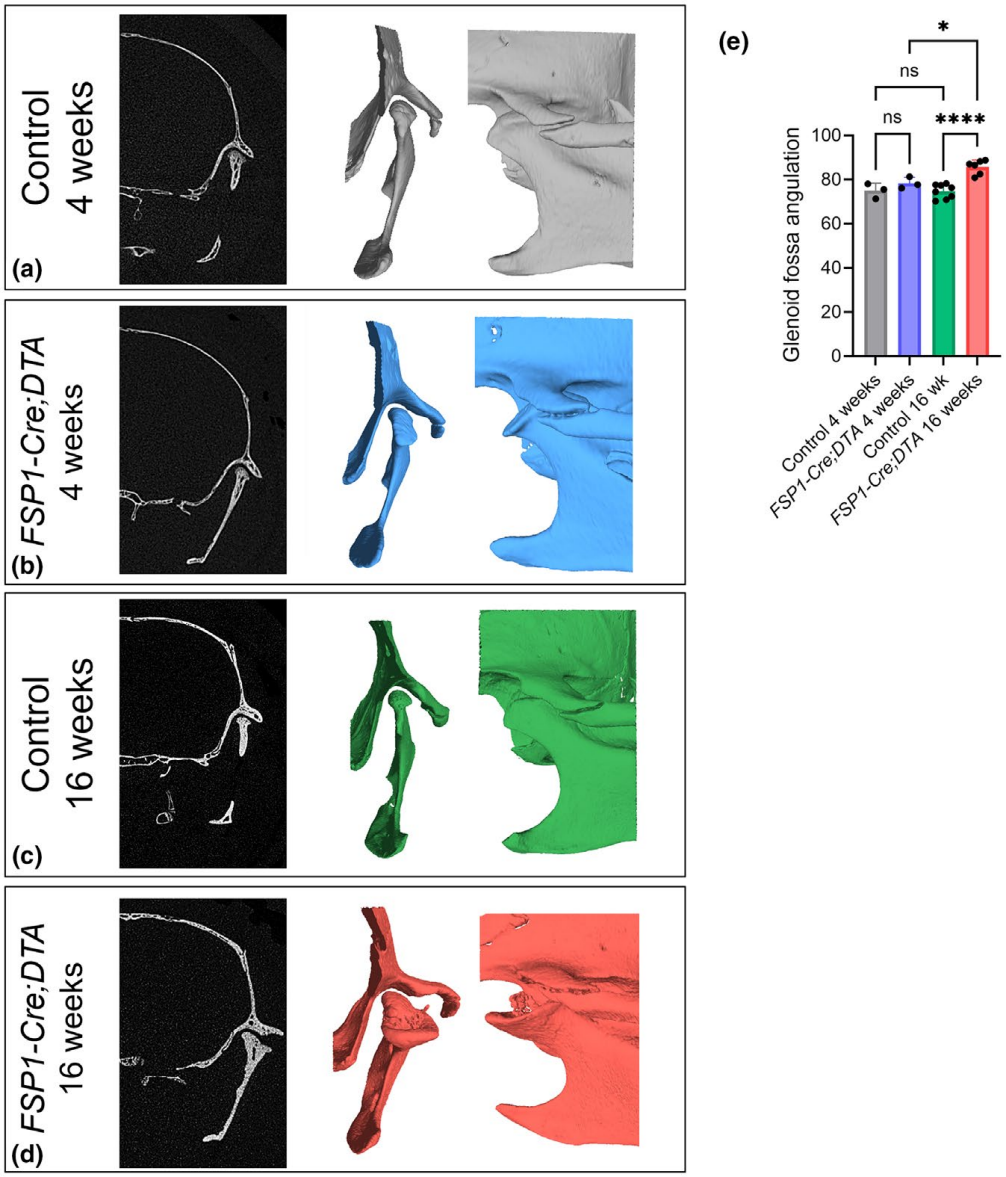
Alterations in the glenoid fossa following removal of superficial layers by FSP1‐Cre‐Driven DTA. (a–d) μCT scans and 3D reconstructions of the TMJ in posterior and lateral views of 4‐week‐old (Data are representative of 3 independent biological replicates) and 16‐week‐old (Data are representative of 6 independent biological replicates) *FSP1‐Cre;DTA* mice, along with Cre‐negative DTA littermate controls 4‐week‐old (Data are representative of 3 independent biological replicates) and 16‐week‐old (Data are representative of 8 independent biological replicates). (e) Measurements of glenoid fossa angulation performed using Amira software. Error bars represent ±SD; statistical analysis was conducted using one‐way ANOVA followed by Tukey's multiple comparisons test (*p < 0.05, ****p < 0.0001).

The distinct molecular signature of the fibrocartilages of the fossa and condyle allowed us to study how defects in one impact the other. For this the effect of disruption of the condyle fibrocartilage on the fossa was assessed using *FSP1‐Cre;DTA* mice. We previously demonstrated that depletion of FSP1‐positive cells resulted in the degradation of condylar structures (Tuwatnawanit et al., 2025). In 4‐week‐old FSP1‐depleted mice the condyle had developed minor changes to its head as viewed by microCT, while the glenoid fossa appeared unaffected (Figure 4a,b) (*n* = 3 mutants and 3 controls). These findings were confirmed by histology (Figure S9A–D) (3/3 mutants). The primary defect was therefore in the condyle and not the fossa, agreeing with the relative contribution of FSP1 expressing cells to these structures. Importantly, by 16 weeks, the glenoid fossa exhibited significant abnormalities and morphological alterations. These changes included enlargement and lateral expansion of the fossa compared to Cre‐negative DTA littermate controls, with changes in the fossa shape linked to enlargement of the condylar head (Figure 4c,d) (*n* = 6 mutants and 8 controls). A pronounced change in the angulation of the fossa was observed, which was significantly wider in the 16‐week‐old mutant mice compared to controls or 4‐week‐old mutants (Figure 4e).

**FIGURE 5:**
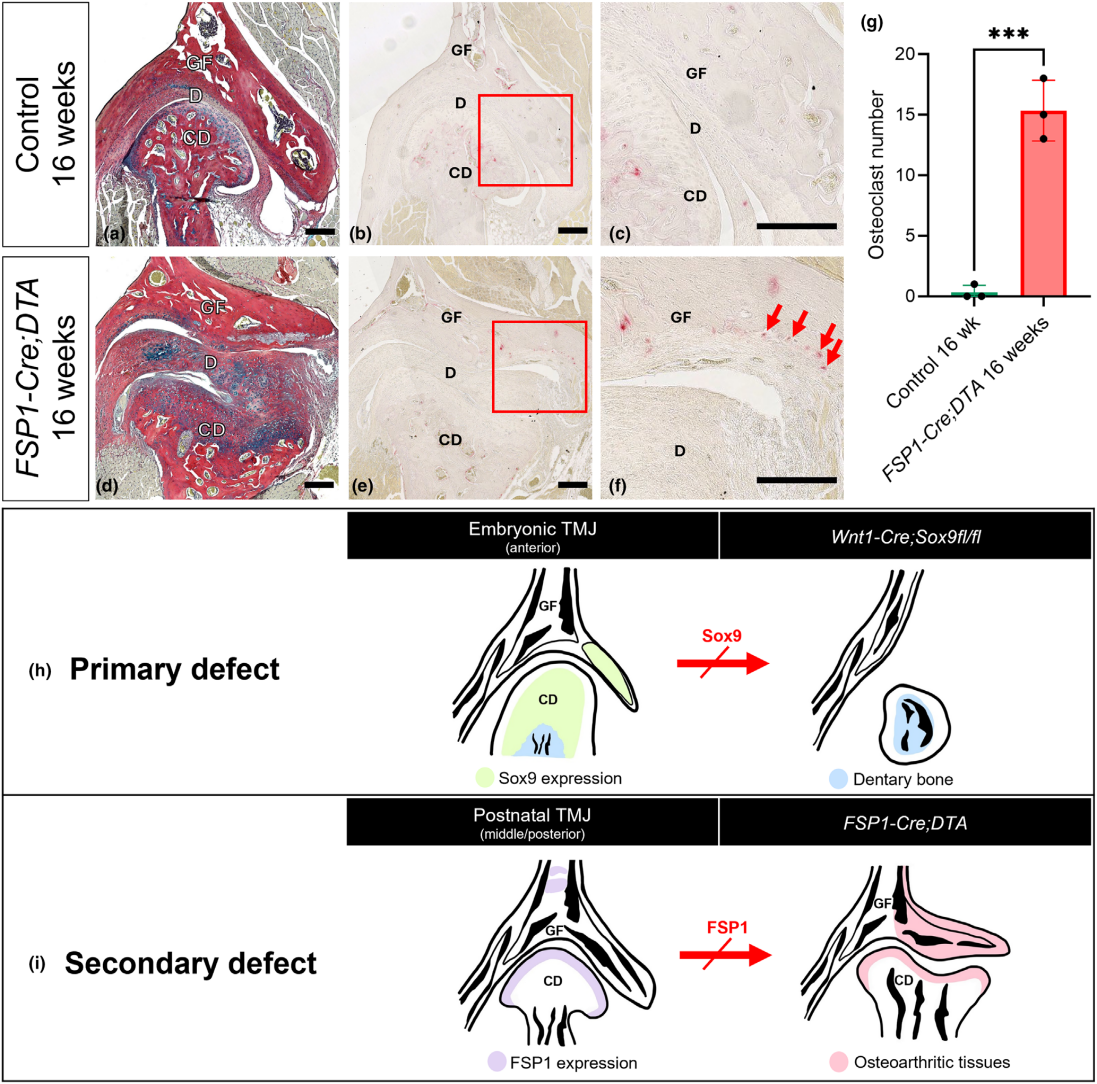
Impact of FSP1‐cell loss on glenoid fossa remodelling activity and schematic representation of glenoid fossa phenotypes resulting from primary and secondary defects. (a, d) Picrosirius red–alcian blue trichrome staining illustrating the TMJ structure (middle to posterior aspect) in 16‐week‐old *FSP1‐Cre;DTA* mice along with the Cre‐negative *DTA* littermate controls. (b, e) TRAP staining of the TMJ structure. The box shows a zoomed‐in image (c, f); arrows indicate osteoclasts (red) in the glenoid fossa surface. Data are representative of three independent biological replicates. Scale bar: 200 μm. (g) The number of osteoclasts on the lateral branch surface of the glenoid fossa in 16‐week‐old *FSP1‐Cre;DTA* mice compared with Cre‐negative DTA littermate controls. Error bars represent ±SD; *n* = 3; statistical analysis was conducted using unpaired *t*‐test. (***p < 0.001). (h) Primary defect during embryonic development: Loss of Sox9 in the lateral branch of the glenoid fossa and the condyle leads to the absence of TMJ structures, including the lateral branch of the glenoid fossa, in *Wnt1‐Cre;Sox9fl/fl* mice. (i) Secondary defect during postnatal development: Loss of FSP1‐expressing cells in the fibrocartilage layer of the condyle results in an osteoarthritis‐like phenotype, characterised by lateral expansion and increased bone remodelling of the glenoid fossa.

As previously described in *FSP1‐Cre;DTA* mice, the condylar surface became disrupted and uneven at 16 weeks (Tuwatnawanit et al., 2025). Here, trichrome staining revealed pronounced transformations in the TMJ structure, culminating in a severe osteoarthritic phenotype not only in the condyle but also in the TMJ disc, which thickened, lost its concave configuration and exhibited signs of ectopic cartilage formation (3/3 mutants) (Figure S9E–H). Agreeing with the microCT findings, the lateral wing of the glenoid fossa flared out to accommodate the changes to the other parts of the TMJ (Figure 5a,d).

Bone remodelling, a crucial process that regulates bone mineral density, plays a significant role in maintaining joint balance and function within the TMJ (Cardoneanu et al., 2023; Tanaka et al., 2008). This process involves osteoclasts breaking down old bone and osteoblasts forming new bone. To assess changes in osteoclast activity in the fossa TRAP staining was performed (Ethiraj et al., 2022). During late prenatal and postnatal development, TRAP‐positive cells were observed in the forming fossa, suggesting that active bone remodelling may be involved in the creation of normal fossa shape (Figure S10). In adult mice, however, only a few TRAP‐positive cells were located in the fossa, with higher numbers associated with the osseous part of the condyle (Figure 5b,c). In contrast, *FSP1‐Cre;DTA* mice exhibited a significant increase in osteoclast activity in the fossa, particularly within the superficial layer near the TMJ disc (Figure 5e–g). These TRAP‐positive cells, actively engaged in bone resorption, displayed distinctive morphological features at the bone tissue surface, characterised by irregular, ruffled or scalloped‐shaped borders in the resorbed area (Figure 5f).

## 4 DISCUSSION

The glenoid fossa has a complex 3D shape with a fibrous connective tissue band between the zygomatic process and the squamosal bone in the anterior region at all stages of development. Posteriorly, these regions converge and eventually fuse, forming a triangular shape. This triangular configuration of the glenoid fossa exhibits distinct morphological characteristics and can be used to determine the position of the condyle along the anterior–posterior axis of the TMJ. Lineage tracing confirmed the glenoid fossa to be derived from neural crest cells.

*Sox9*‐expressing cells serve as progenitors for both chondrogenic and osteogenic lineages, including those involved in intramembranous ossification (Akiyama et al., 2005; Anthwal et al., 2015). The transient expression of *Sox9* in progenitor cells is not maintained in cells undergoing intramembranous ossification with *Sox9* downregulated in cells committing to the osteoblast lineage (Mori‐Akiyama et al., 2003). Here, we show that Sox9 was transiently expressed in the mesenchymal condensation of the lateral branch of the glenoid fossa at E14.5. The lateral branch of the glenoid fossa has been reported to express Col‐X, suggesting that a cartilage template may play a role in its development (Purcell et al., 2012). However, our study did not detect Col‐X expression in this region. In the *Sox9* conditional ablation model using *Wnt1‐Cre;Sox9fl/fl* mice, both the lateral branch of the glenoid fossa and the condyle were absent, highlighting the importance of *Sox9* in the initiation of both these structures. Interestingly, previous research using the same mutant, showed that the lateral branch of the glenoid fossa bone formed did not fuse to the rest of the fossa, while our mutants showed no lateral branch, suggesting differences due to mouse background (Wang et al., 2011). As Sox9 was expressed transiently in the lateral branch we conclude that defects in the fossa were primary, rather than secondary (Wang et al., 2011) (Figure 5h). The expression of Sox9 only in the lateral branch of the forming fossa suggests subtle differences in development between the medial and lateral sides, which might reflect a different evolutionary history.

The condyle forms as a secondary cartilage associated with the dentary bone during prenatal development, and its initiation is not dependent on mechanical load (Anthwal et al., 2008, 2015; Rot‐Nikcevic et al., 2007). In contrast, the fibrocartilage layer of the glenoid fossa formed postnatally, correlating with the completion of tooth eruption (Lungová et al., 2011), suggesting a potential role for mechanical force in its initiation. Fibrocartilage is described as a transitional tissue that undergoes significant developmental and maturation changes in its composition, organization and cell characteristics over time (Benjamin & Ralphs, 2004; Park et al., 2015). This distinction in the developmental timeline may be linked to the unique physiological and functional characteristics of the glenoid fossa. Unlike the mandibular condyle, the glenoid fossa exhibits distinct responses to mechanical loading and forms chondroid bone around P21, coinciding with the transition of pups from a liquid to a solid diet (Yasuda et al., 2012). The adaptiveness of the joint to mechanical loads and stress allows the fibrocartilaginous tissues to respond to changes in dietary habits and functional demands during postnatal growth (Nagy & Daniel, 1992). The secondary cartilage of the fossa, therefore, may be more similar to the mechanically induced secondary cartilages observed in birds (Solem et al., 2011) than the secondary cartilage of the condyle.

The fibrocartilage layers of the mature condyle predominantly express FSP1, with relatively low levels of *Axin2* (Tuwatnawanit et al., 2025). In contrast, during postnatal development of the fibrocartilage layer of the glenoid fossa, *Axin2* expression was shown to be high, while FSP1 levels were low, confirmed by following the lineage of FSP1 cells from development to adulthood. These findings highlight distinct molecular profiles and regulatory mechanisms governing the formation and maturation of the fibrocartilage in the two articular surfaces of the TMJ. This fits with the different developmental processes responsible for the formation of the condyle (secondary cartilage) and fossa (intramembranous bone). The TMJ (or dentary‐squamosal joint) is a mammalian novelty, created by the coming together of two intramembranous bones. During evolution, therefore, a fibrocartilage cap to line the new articulation would have needed to be added. In the lower jaw this was created by the initiation of a secondary cartilage, the condyle. Our analysis suggests that the fibrocartilage of the fossa might have been added later using a different mechanism in response to force.

During both development and homeostasis, it is well established that defects in one component of the TMJ can influence other interconnected structures (Li et al., 2021; Wang et al., 2011). In *FSP1Cre;DTA* mice, diphtheria toxin expression in *Fsp1*‐expressing cells led to the loss of the condylar fibrocartilage layer, resulting in a severe TMJOA phenotype and an enlarged condylar head (Tuwatnawanit et al., 2025). Interestingly, ectopic chondrogenesis was observed along the surface of the condyle and within the TMJ disc, which we interpret as a maladaptive response aimed at stabilizing joint articulation and maintaining structural proximity. In contrast to the emerging defects in the condyle, the fossa showed a normal morphology and structure at 4 weeks, in keeping with the low expression of FSP1 in the fossa. In contrast by 16 weeks, substantial changes in the shape and articular surface of the glenoid fossa were observed. These changes allowed the fossa to encircle the expanding condylar head, keeping close contact between the two articulating surfaces to preserve joint function. Although some FSP1 expression was observed in the forming bone of the fossa, which would be impacted in these mutants, the changes at the articulation, where FSP1 is not expressed, are likely to be a secondary consequence of defects in the condyle (Figure 5i).

The changes in the fossa in both the *Sox9* mutants and *FSP1* mutants were more pronounced on the lateral side, despite these two phenotypes being generated by very different mechanisms (Figure 5h,i). The convergence of the defects driven by changes to distinct pathways, suggests that the lateral branch is particularly vulnerable to defects. Such vulnerability has been linked to structures that are more amenable to change over evolution, with a trade‐off between a higher capacity for innovation and a higher propensity for errors/dysfunction (Hallgrimsson et al., 2019). The defects focused on the lateral branch could reflect differences in responsiveness to mechanical force or differences in the ability to adapt due to location, with expansion on the medial side restricted due to the closeness to the brain. The lateral branch of the fossa may have evolved later during the evolution of the mammalian TMJ to stabilise the fledgling joint and increase the contact area between the upper and lower jaw. This fits with the hypothesis that later forming and evolving structures show higher variation, as observed in the limb (Sears et al., 2018).

The change in fossa shape was linked to an increase in TRAP‐positive osteoclasts on the glenoid fossa surface, indicating heightened bone remodelling during disease progression. This agrees with prior studies that have suggested that degenerative changes in TMJOA may result from improper remodelling due to prolonged or excessive joint loading and a diminished adaptive capacity (Cardoneanu et al., 2022; Tanaka et al., 2008). In TMJOA, bone remodelling is typically characterised by a reduction in both osteoblast quantity and functionality, accompanied by increased osteoclast activity (Embree et al., 2011). Thus, the *FSP1‐Cre;DTA* mouse model provides a valuable system for exploring the effects of condyle fibrocartilage loss on the neighbouring tissues. Additionally, it offers important insights into TMJ adaptation and TMJOA pathology, making it a useful tool for studying joint degeneration and potential therapeutic strategies.

## AUTHOR CONTRIBUTIONS

AST and NA conceptualised the work; AST, NA and TT designed the experiments. TT and NA performed the experiments and analysed the data. TT, NA and DB performed tissue collection. TT wrote the original manuscript. NA, AST, ZSK and DB edited the manuscript. AST, NA and ZSK acquired funding and supervised the work. All authors gave their final approval and agreed to be accountable for all aspects of the work.

## CONFLICT OF INTEREST STATEMENT

Prof Tucker is currently President‐Elect of the Anatomical Society. The other authors declare no conflict of interest.

## Supporting information


Data S1.

## Data Availability

The data that support the findings of this study are available in the supplementary material of this article.
