# Supplementary material for: Mapping the murine TMJ glenoid fossa over development, homeostasis and in response to disease
Source: J Anat. 2025 Nov 10;249(1):85–96. doi: 10.1111/joa.70068 (PMC13238677; doi:10.1111/joa.70068)
Supplement: Supplementary file 1 — Data S1. [file JOA-249-85-s001.docx]

**SUPPLEMENTARY**


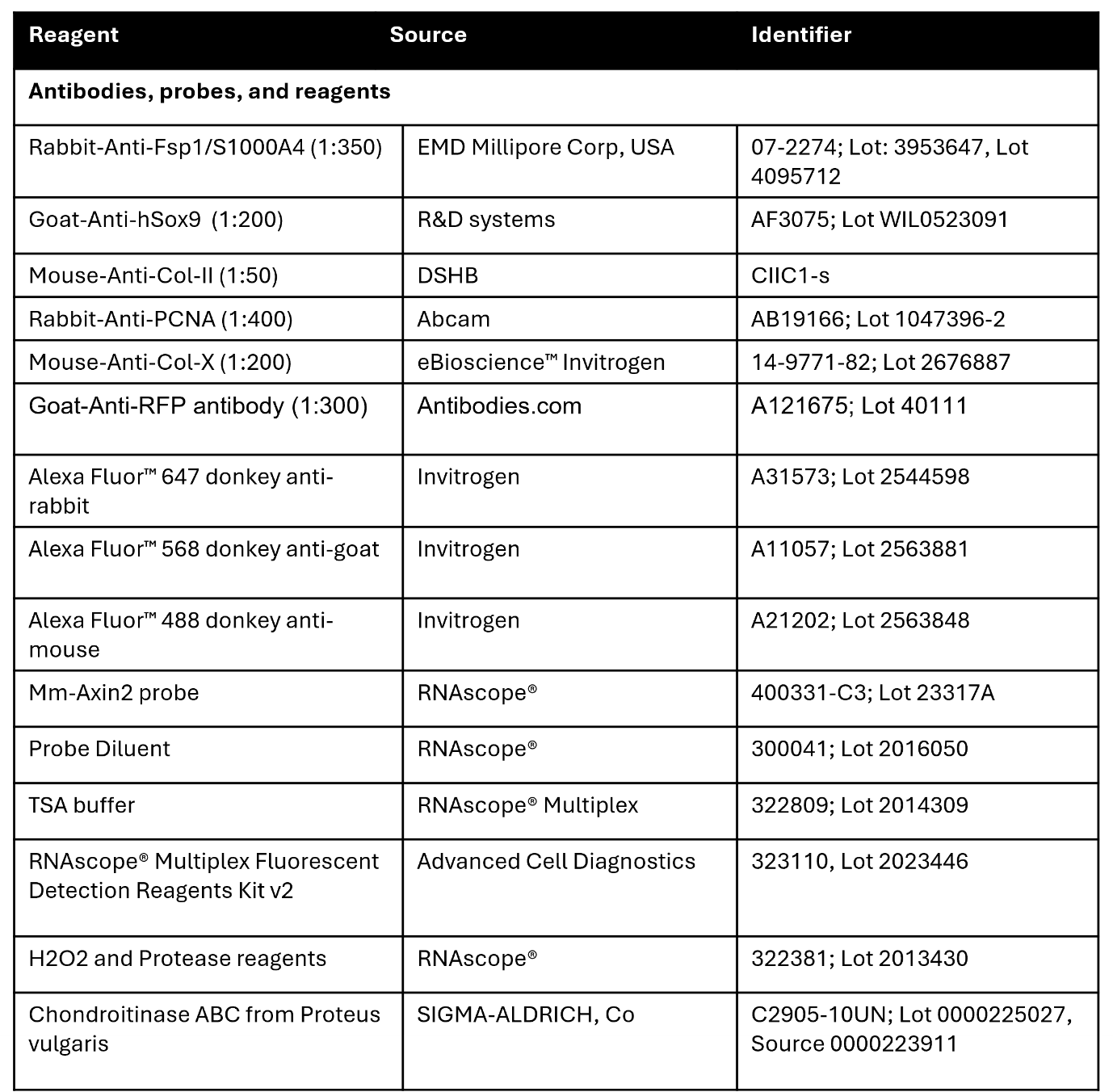


**Table supplementary 1: Antibodies, probes, and reagents**


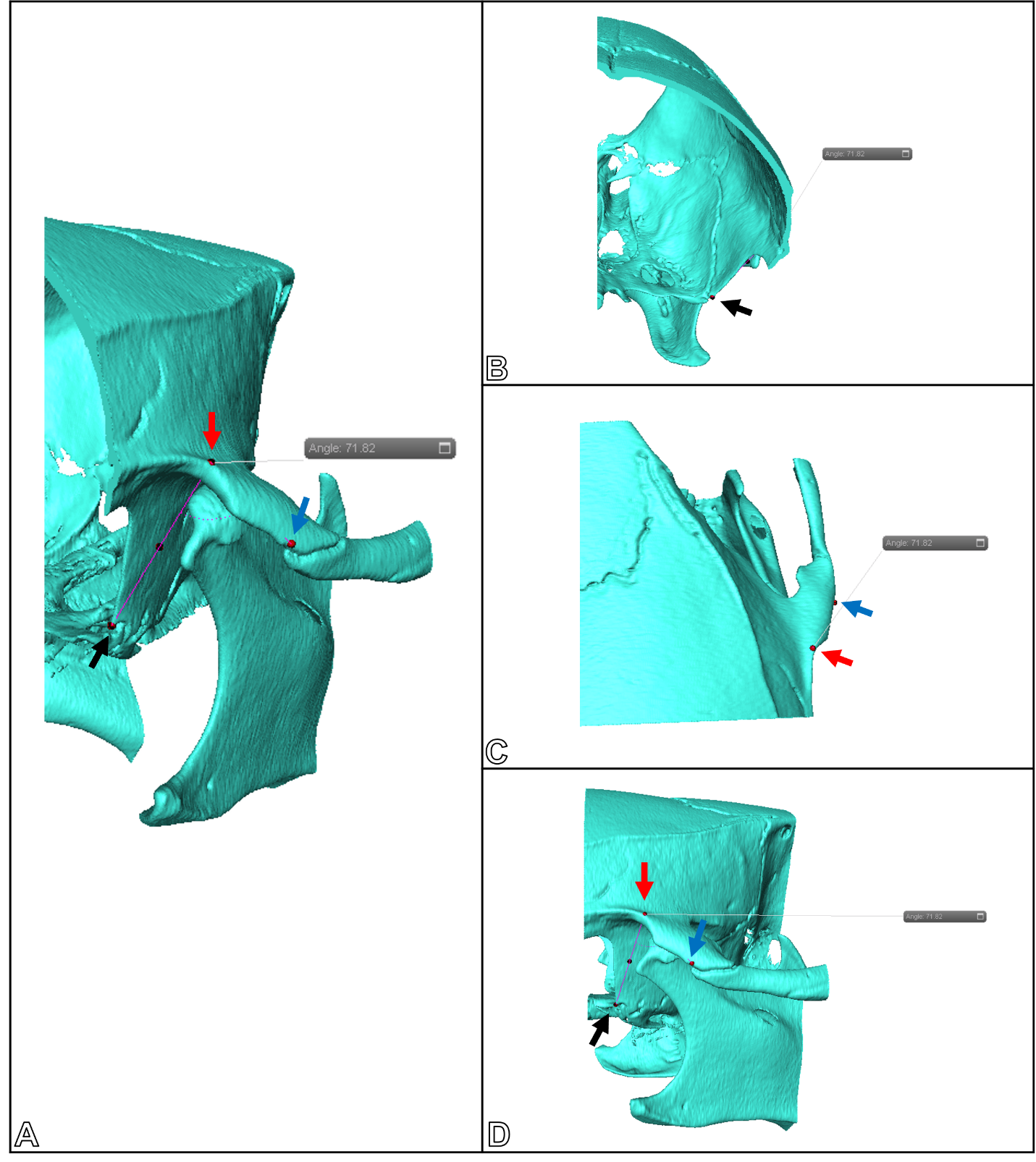


**Figure supplementary 1: Glenoid fossa angulation landmarks**

(A-D) 3D reconstruction µCT scans of the TMJ in different views in a 16-week-old *FSP1-* Cre-negative *DTA* control. Black arrow shows the intersection of the squamosal, auditory capsule, and greater wing of the sphenoid bone. Red arrow shows the joining of the squamosal body to the zygomatic process of the squamosal. Blue arrow shows the posterior extent of the zygoma (jugal) where it intersects with the zygomatic process of the squamosal (squamosal-zygomatic suture, or temporozygomatic suture in humans).


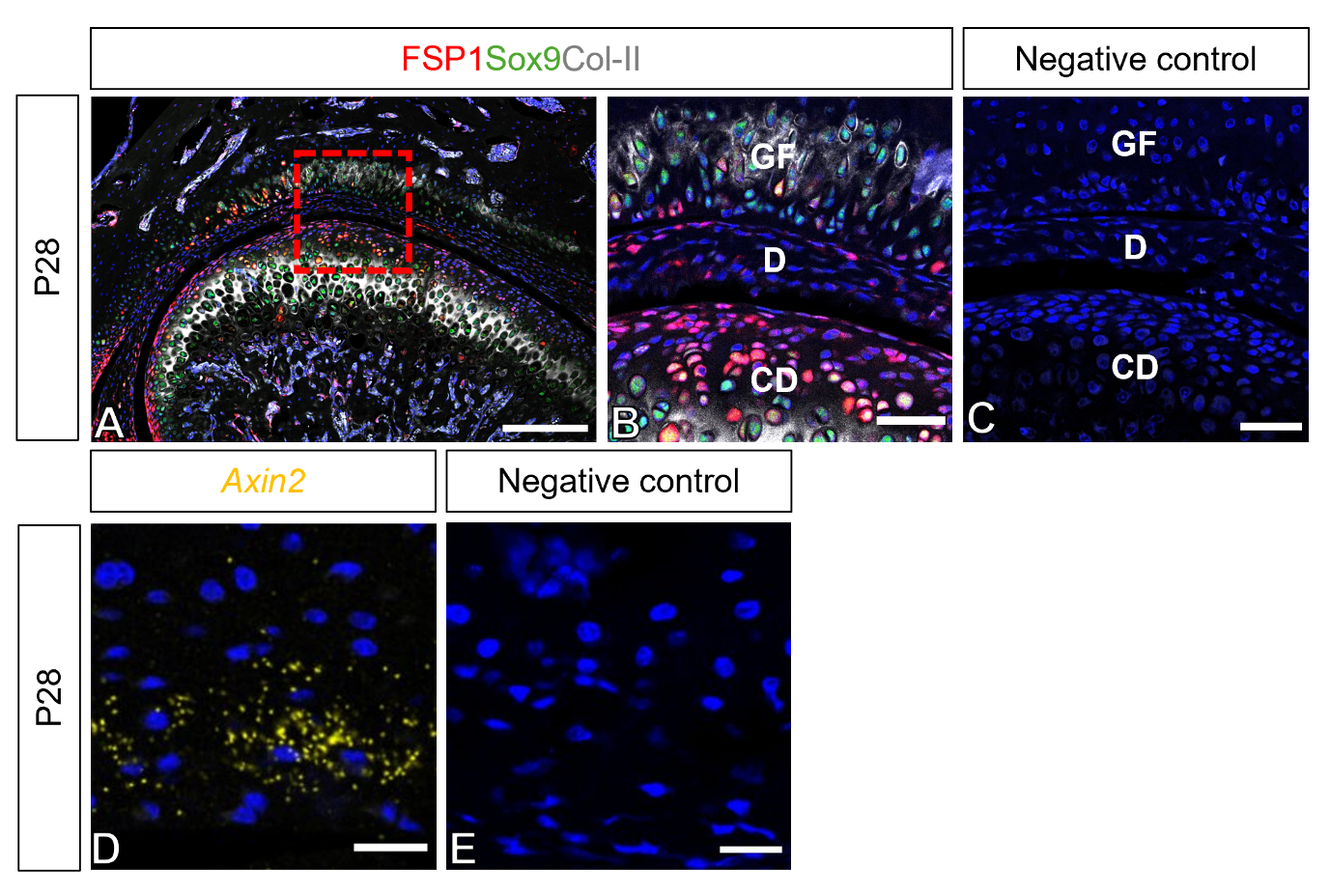


**Figure supplementary 2: Immunofluorescence and RNAscope control slides.**

(A) Immunofluorescence staining for FSP1 (red), Sox9 (green), Col-II (grey), and DAPI (blue) in P28 CD1 mice. (B) The box shows a zoomed-in image of figure A. (C) Negative control slide of immunofluorescence staining in A. (D) RNAscope staining for *Axin2* mRNA (yellow) and DAPI (blue) in P28 CD1 mice. (E) Negative control slide of RNAscope staining in D. Scale bar A: 200 µm; B-C 50 µm; D-E 20 µm.


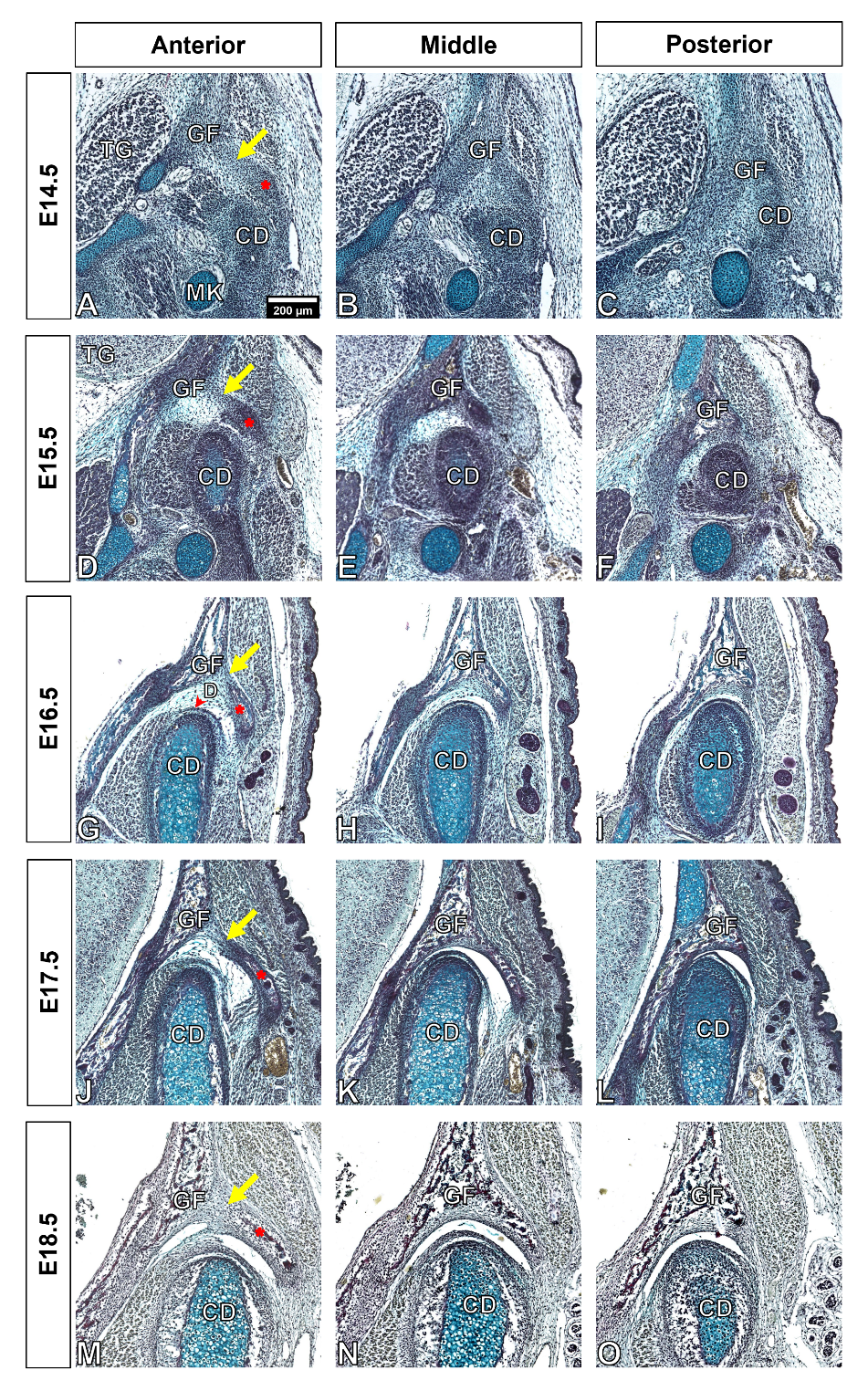


**Figure supplementary 3: Development and growth of the mouse TMJ during embryonic stage**

(A-O) Picrosirius red–alcian blue trichrome staining illustrating the development of the TMJ at E14.5 (A-C), E15.5 (D-F), E16.5 (G-I), E17.5 (J-L), and E18.5 (M-O) in CD1 mice (n=5). Each stage includes images showing the anterior, middle, and posterior aspects of the TMJ in frontal sections. The yellow arrow points to a fibrous tissue band between the zygomatic process and the squamosal bone. The red asterisk marks the lateral branch of the glenoid fossa. (G) The red arrowhead points the TMJ disc. The scale bar represents 200 µm. The scale bar in A applies to B-O. CD, condyle; D, TMJ disc; E, embryonic stage; GF, glenoid fossa; MK, Meckle’s cartilage; TG, Trigeminal ganglion.


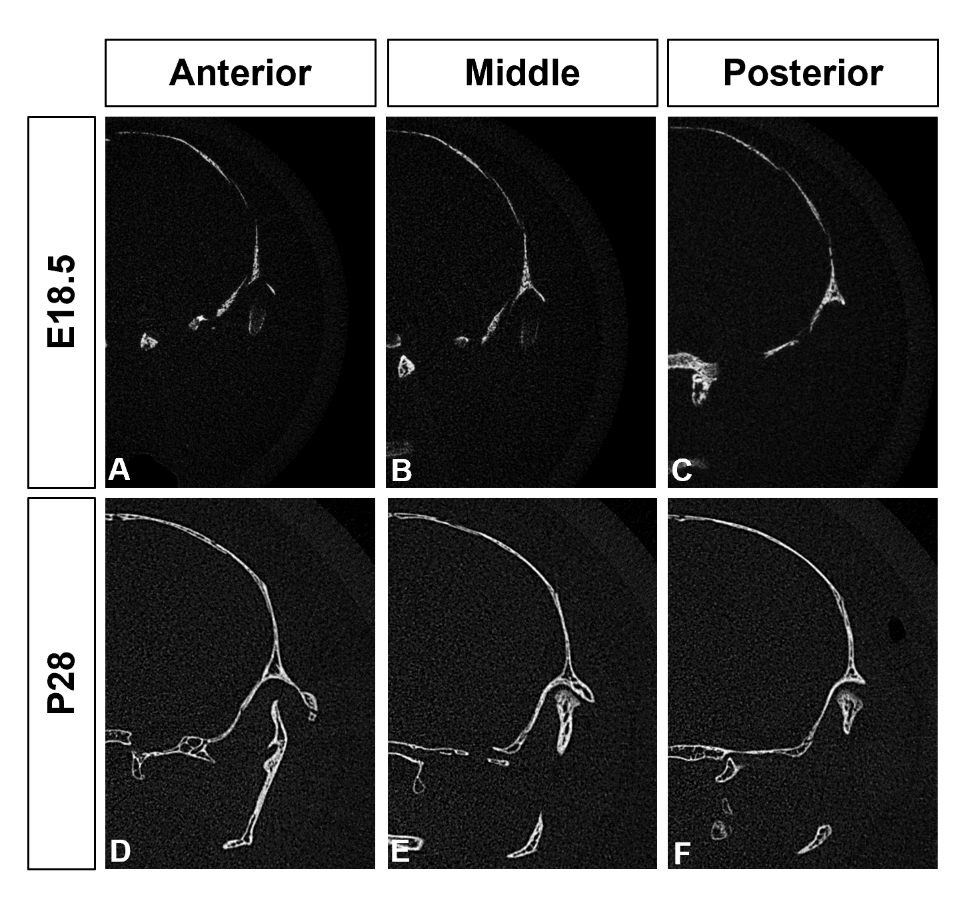


**Figure supplementary 4. MicroCT scan of the mouse TMJ glenoid fossa.**

(A-F) μCT scans of the TMJ glenoid fossa in anterior, middle, and posterior aspects at E18.5 and P28 in CD1 mice.


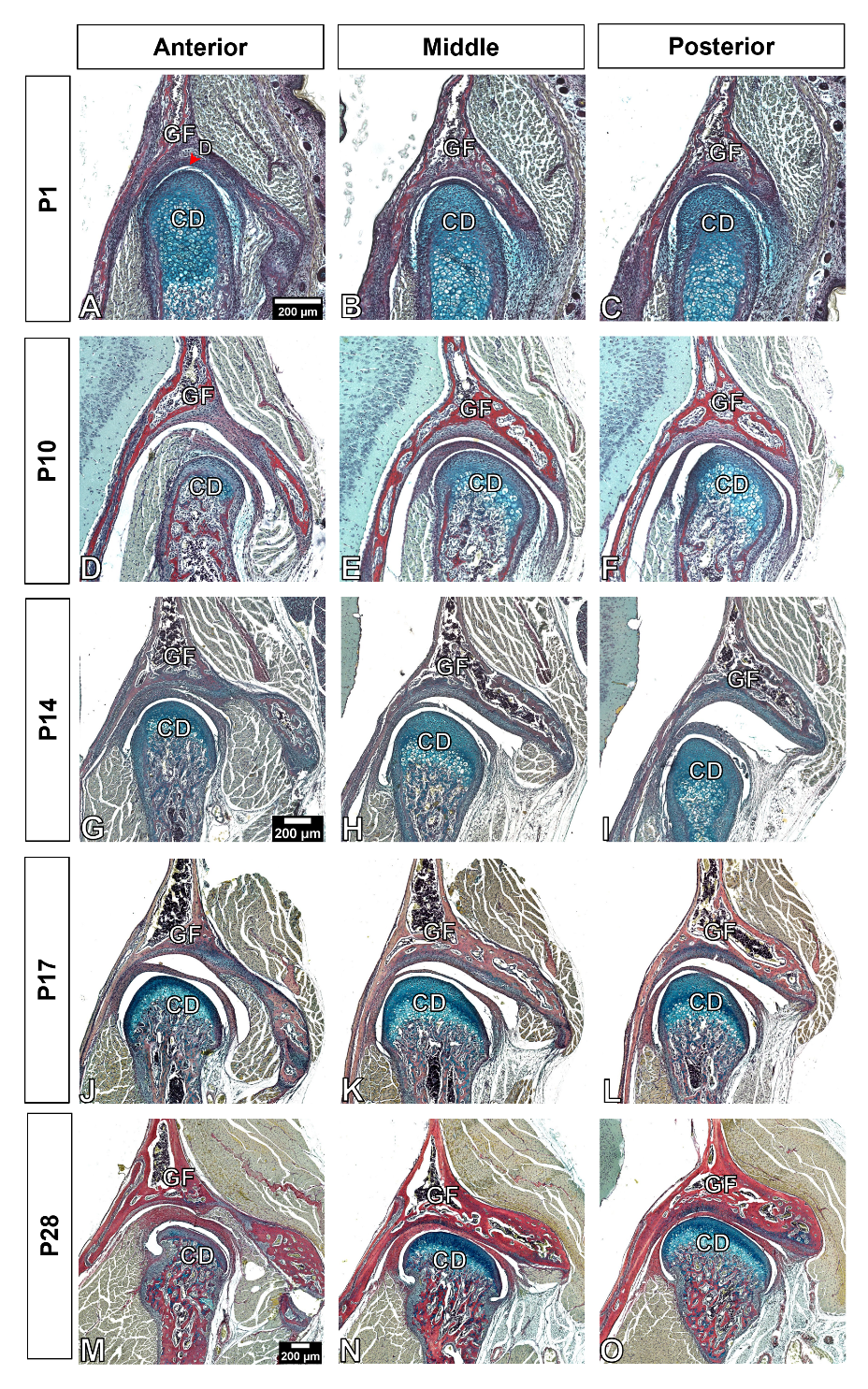


**Figure supplementary 5: Development and growth of the mouse TMJ during postnatal stage**

(A-O) Picrosirius red–alcian blue trichrome staining illustrating the development of the TMJ at P1 (A-C), P10 (D-F), P14 (G-I), P17 (J-L), and P28 (M-O) in CD1 mice (n=5). Each stage includes images showing the anterior, middle, and posterior aspects of the TMJ in frontal sections. The scale bar represents 200 µm. The scale bar in A applies to B-F. The scale bar in G applies to H-I. The scale bar in M applies to N-O. CD, condyle; P, postnatal stage; GF, glenoid fossa.


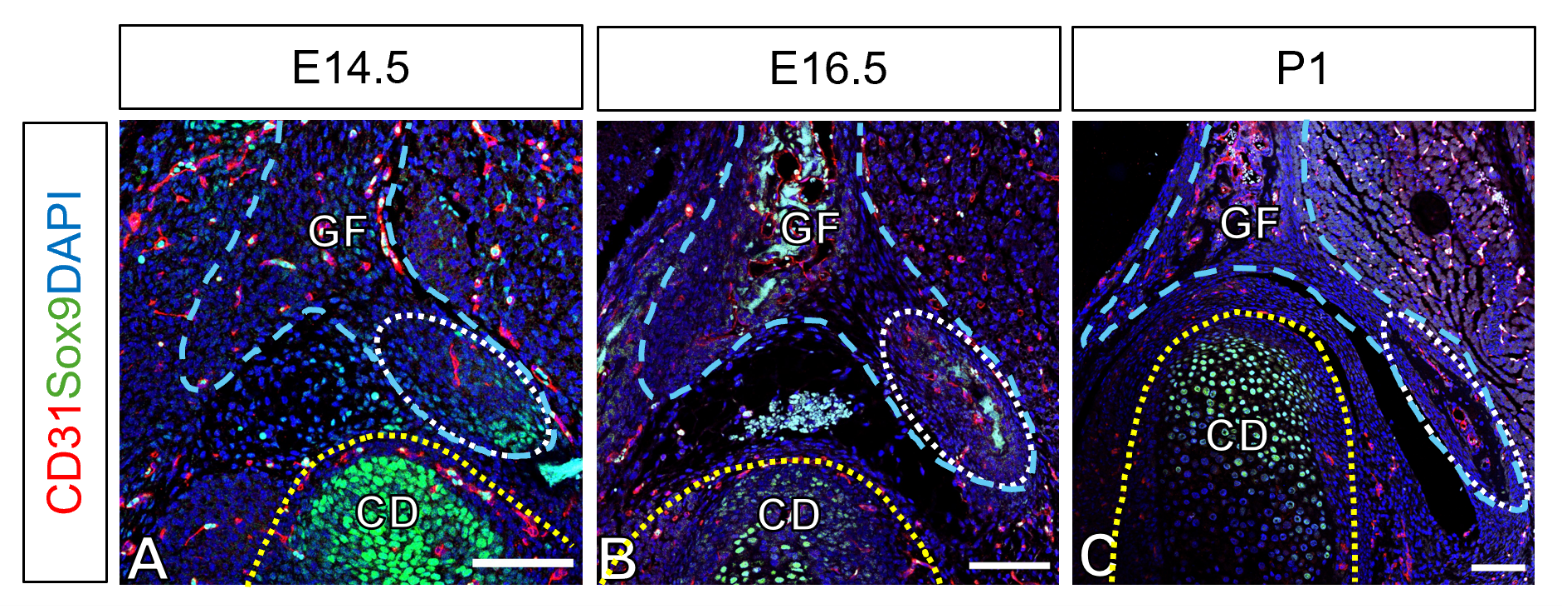


**Figure supplementary 6:**

**Transient Sox9 expression at lateral branch of the glenoid fossa is not associated with the vasculature**

(A) E14.5, (B) E16.5, and (C) P1 CD1 mice. Immunofluorescence staining for endothelial marker CD31 (red), Sox9 (green), and DAPI (blue). Each stage includes images showing the anterior aspects of the TMJ in frontal sections. The yellow dotted line indicates the condylar head. The cyan dotted line indicates the glenoid fossa region with the white circle marking its lateral branch. (A-C) Sox9 was highly expressed in the condyle at all three stages. (A) At E14.5, Sox9 was observed in the mesenchymal condensation of the lateral branch of the glenoid fossa, and Sox9-positive cells were confirmed to be absent from the blood vessel of the lateral branch. (B,C) At later stages Sox9 expression was lost in the lateral branch of the fossa.


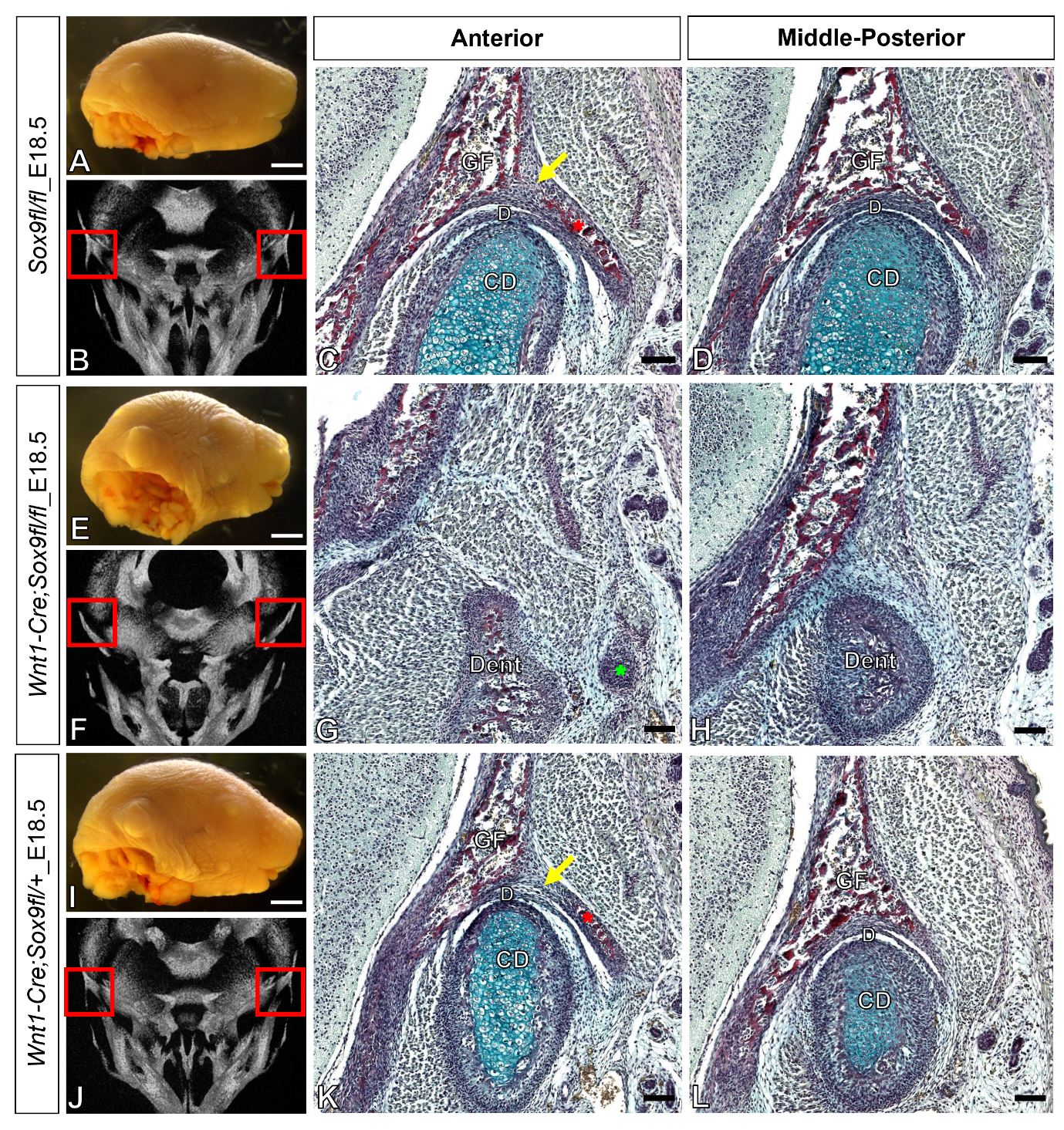


**Figure supplementary 7: Conditional knockout of *Sox9* in neural crest-specific cells resulted in the absence of the glenoid fossa.**

(A) *Sox9f/f* mice (control) at E18.5 (n=3). (B) μCT scan of the skull of *Sox9f/f* mice with the red box highlighting the TMJ region. (C,D) Picrosirius red–alcian blue trichrome staining illustrating the TMJ at E18.5 in control mice, including images showing the anterior and middle-to-posterior aspects of the TMJ in frontal sections. (E) *Wnt1-Cre;Sox9f/f* mice (mutant) at E18.5 (n=3). (F) μCT scan of the skull of *Wnt1-Cre;Sox9f/f* mice with the red box highlighting the absence of the glenoid fossa. (G,H) Picrosirius red–Alcian blue trichrome staining illustrating the absence of TMJ formation in mutant mice compared to the parallel TMJ region in littermate controls at E18.5, including images showing the anterior and middle-to-posterior aspects in frontal sections. (I) *Wnt1-Cre;Sox9f/+* mice (heterozygous) at E18.5 (n=3). (J) μCT scan of the skull of *Wnt1-Cre;Sox9f/+* mice with the red box highlighting the TMJ region, showing less radiopacity at the lateral branch of the glenoid fossa compared to littermate control mice. (K,L) Picrosirius red–alcian blue trichrome staining showing a thinner lateral branch of the glenoid fossa in *Wnt1-Cre;Sox9f/+* mice compared to littermate controls at E18.5, including images showing the anterior and middle-to-posterior aspects of the TMJ in frontal sections. (C,K) The yellow arrow points to a fibrous tissue band between the zygomatic process and the squamosal bone. (C,K) The red asterisk marks the lateral branch of the glenoid fossa. (G) The green asterisk marks the zygomatic arch. Scale bars: A,E,I 5mm; C-D,G-H,K-L 100 µm. Dent, dentary bone.


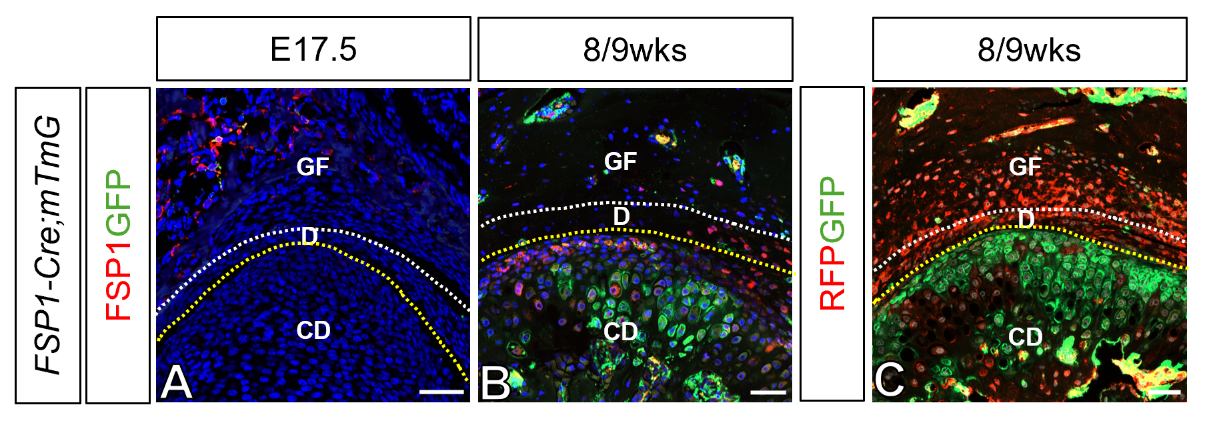


**Figure supplementary 8: Expression and contribution of FSP1-expressing cells in the mouse TMJ during embryonic and postnatal stages.**

(A-C) Lineage tracing analysis in *FSP1-Cre;mTmG* mice. (A-B) Immunofluorescence staining for FSP1 (red) shows current FSP1 expression with lineage tracing of these cells (GFP, green), and DAPI (blue) in the TMJ at E17.5 (A) and in the adult (specimens analysed at 8 and 9 weeks) (B). (C) Immunofluorescence staining confirming lineage tracing of FSP1-positive cells (GFP, green) and FSP1-negative cells (RFP, red) in the TMJ at 8 and 9 weeks. Scale bar: 50 µm. E, embryonic stage; FSP1, fibroblast specific protein 1; GFP, green fluorescent protein; RFP, red fluorescent protein. CD = condyle, D = disc, GF = Glenoid Fossa.


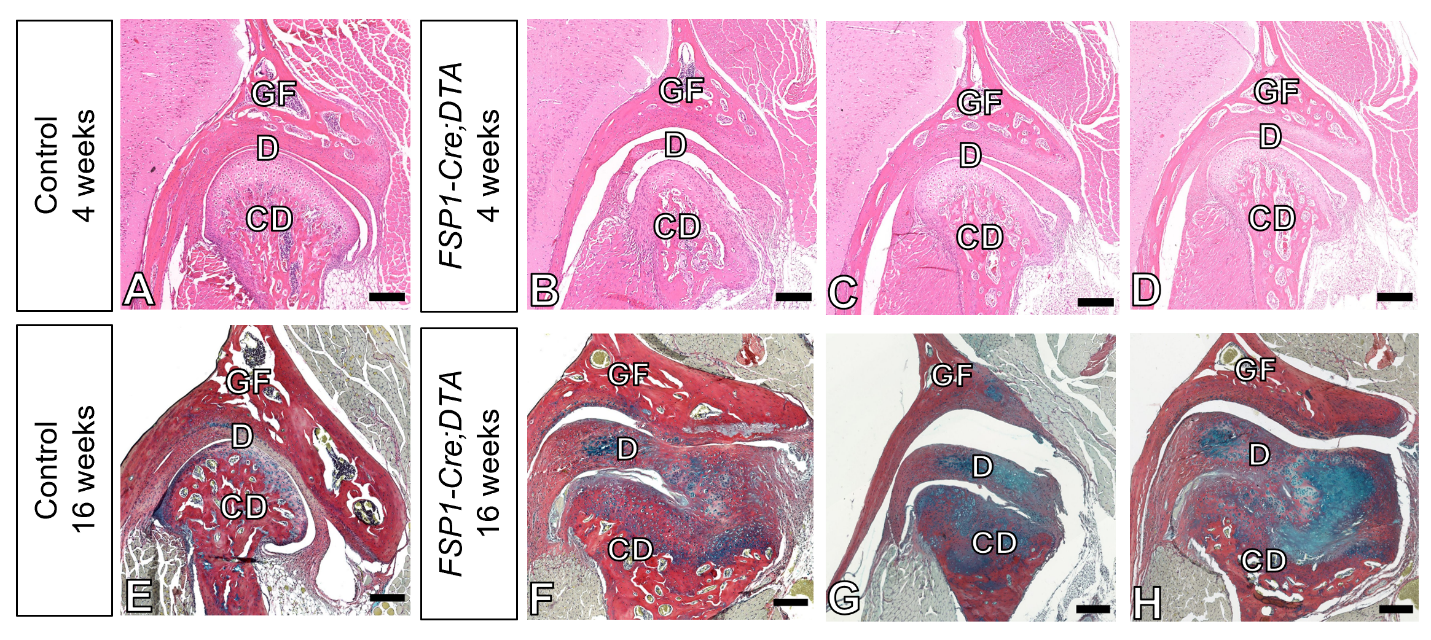


**Figure supplementary 9: Histological changes in mutant TMJs.**

**(A–D)** H&E staining of 4-week-old *FSP1-Cre;DTA mice*, compared with Cre-negative DTA littermate controls, revealed altered condyle and disc morphology in the mutant group, while the fossa appeared normal at this stage. **(E-H)** Picrosirius red–alcian blue trichrome staining illustrating the TMJ structure (*E*, *F*, *H*: middle to posterior TMJ; *G*: very posterior TMJ) in 16-week-old *FSP1-Cre;DTA* mice and Cre-negative *DTA* littermate controls. Mutant mice exhibit a severe osteoarthritic phenotype. The disc is thickened, loses its normal concave–convex configuration, and displays signs of ectopic cartilage formation. Data represents three independent biological replicates. Scale bar: 200 µm.


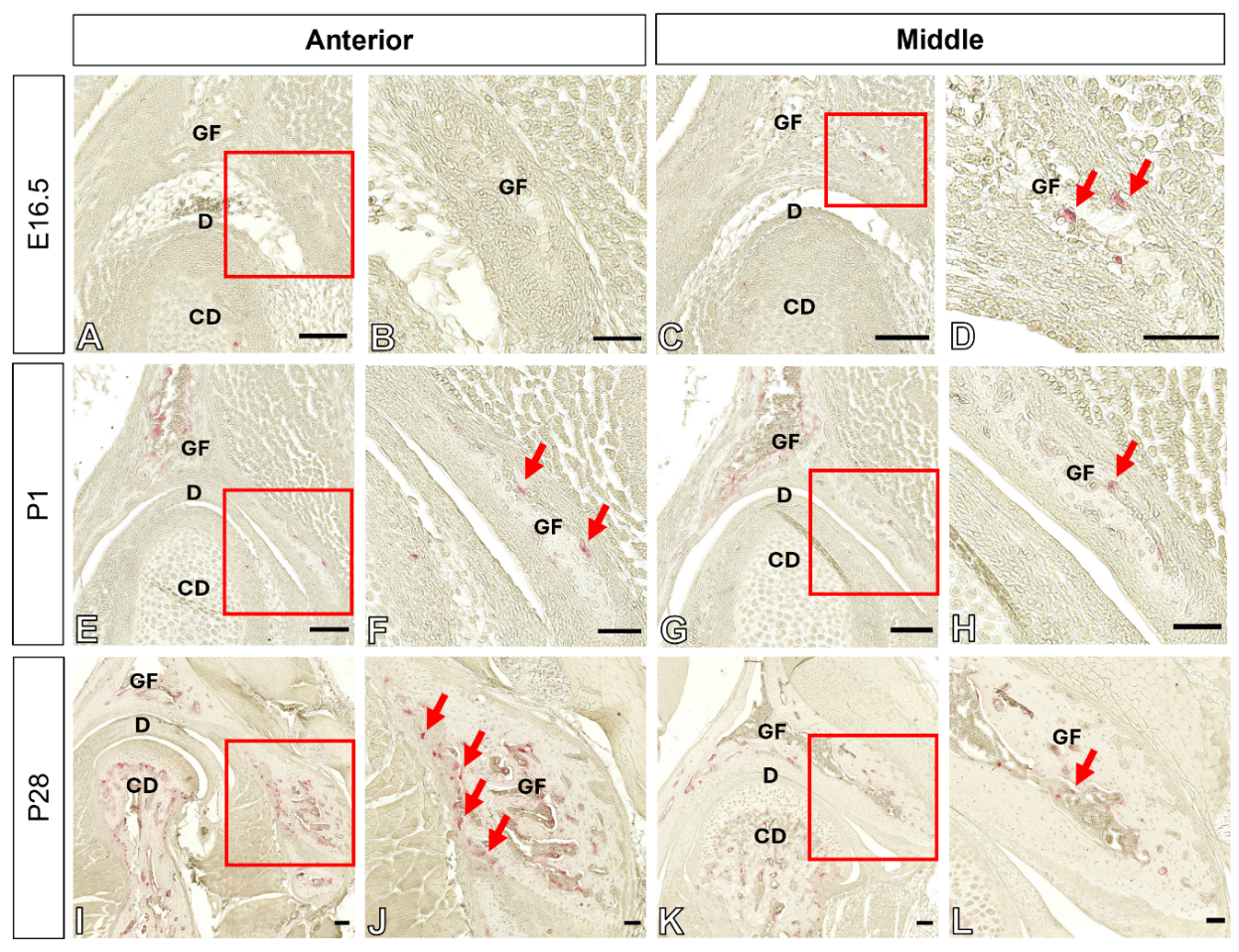


**Figure supplementary 10: Osteoclast activity in the glenoid fossa during TMJ development**

(A-L) TRAP staining illustrating the development of the TMJ during embryonic stages at E16.5 (A-D), and during postnatal growth at P1 (E-H) and P28 (I-L) in CD1 mice (n=3). Each stage includes images showing the anterior and middle aspects of the TMJ in frontal sections. The box shows a zoomed-in image; arrows indicate osteoclasts (red) in the glenoid fossa. Scale bar in A,C,E,G,I,K 100 µm; B,D,F,H,J,L 50 µm.
